# No Medical Student Left Behind: A Study to Examine the Effects of Structured Step 1 Support within our Curriculum

**DOI:** 10.15694/mep.2019.000171.1

**Published:** 2019-09-12

**Authors:** Arlene Sachs, Blake Barker, Angela Mihalic, Dorothy Sendelbach, Carol Wortham, Robert Rege

**Affiliations:** 1 University of Texas Southwestern Medical School

**Keywords:** USMLE – Step 1, teaching and learning, educational strategies

## Abstract

This article was migrated. The article was marked as recommended.

**Introduction:**The University of Texas Southwestern Medical School (UT Southwestern) developed a required, pass/fail Step 1 preparation course in conjunction with their new curriculum to support students with structure, encouragement, and accountability. This study examined the Step 1 performance of students who have taken the prep course after completing pre-clinical coursework and factors which predicted outcomes.

**Methods:** Data were collected between January 2017 and July 2018 from 453 2 ^nd^-year medical students enrolled in the six week course. Multilinear regression and Chi-squared analysis were performed to compare pre-clinical course performance with Step 1 readiness and outcomes.

**Results:** Average course final exam scores and CBSSA results were significantly and directly associated with Step 1 scores. Pre-clinical exam performance, practice exam scores at the beginning of the Step 1 preparation period and an increase in practice exam scores over the study period correlated with Step 1 performance. The percent pass rates and mean scores in 2017 were at 97% and 235, but in 2018, no student failed Step 1 (100% pass rate), and the mean score rose to 239.

**Discussion and Conclusion:** A structured Step 1 Prep Course was well-accepted by students. Exam performance can be predicted from pre-clerkship performance and progress made during the course on practice exams and early intervention for at-risk students can improve performance of at-risk students.

## Introduction

The United States Medical Licensing Examination (USMLE) Step 1 exam is the first and arguably most important medical licensure exam in the United States for obtaining a desired residency position ( McCaskill *et al.*, 2007; Jarou, Davis, Kellogg, 2018; Burk-Rafel, Santen, and Purkiss, 2017). The possibility of not attaining a competitive score creates deep concern among medical students because of the weight it holds for residency programs as they prioritize candidates for interviews ( Gauer and Jackson, 2017; Schwartz *et al.*, 2018; Federation.., 2019; Strowd and Lambors, 2010). Although the prudence of using this exam for residency interview screening has been questioned, Step 1 is the only common factor available at the time of residency application for every medical student ( McGaghie, Cohen, Wayne, 2011; Guffey *et al.*, 2011). When faced with the daunting task of choosing students for interviews, residency directors seek the most expedient tool available to cull applications ( Carroll, 2017; Prober *et al.*, 2016).

Therefore, most medical schools provide support and dedicated time for students to prepare for the Step 1 exam. Typically, students develop their own study plan to take Step 1 ( Schwartz *et al.*, 2018) or schools provide student-run Step 1 guidance programs ( Alcamo *et al.*, 2010; Johnson, Jordan and Silbiger, 2013). At Albert Einstein School of Medicine, a “near-peer approach” was found to be beneficial for studying for Step 1 ( Johnson, Jordan and Silbiger, 2013). Another student-initiated approach, developed by Schwartz, *et al.* (2018) included live review sessions led by high-performing upper classmen who assigned reading homework from *First Aid for Step 1* and *UWorld* to be completed before lecture sessions. This approach at the University of Illinois College of Medicine in Chicago raised mean USMLE Step 1 exam scores by 8.82 points and first attempt pass rates by 8%.

UT Southwestern Medical School (UT Southwestern) admits approximately 230 student per year. The current pre-clinical phase transitioned in August of 2015 from a classic 24-month, departmentally-based curriculum to 18 months of pass/fail courses with topics integrated by concepts and organ systems. The pre-clinical curriculum does not overly emphasize preparation for the USMLE Step 1 exam and students are encouraged to focus on course work as a foundation for success with both Step 1 and the clinical phase of their education. The Medical School developed a required, pass/fail Step 1 preparation course (“Step 1 Prep Course”) as part of a revised curriculum implemented in August 2015. This study examines whether the current curriculum and Step 1 Prep Course at UT Southwestern is as effective as our previous curriculum in preparing students for Step 1, and identifies factors that correlate with success on the examination.

## Methods

***Course design:*** The six-week Step 1 Prep Course is offered to second-year students via 5 blocks in the first semester (January to June) of the clinical phase. The Step 1 Prep Course has been required since 2017, and is based on a 2016 pilot version offered in the previous curriculum. The Director of Student Academic Support Services (SASS), an experienced educational advisor, serves as course director. At-risk students, who perform poorly in their pre-clinical courses or who have wellness concerns, are advised to request clerkship schedules easily adapted for additional Step 1 study time; other students make choices based on personal preferences. Thus, placement into the prep course blocks is neither random nor based on student preference alone.

All students are invited to workshops to enhance understanding of the preparation process, and to cover topics such as time management, development of personal study plans, relative benefits and efficient usage of study resources, and exploration of basic strategies for stress management and overall wellness. They must take a school-provided NBME Comprehensive Basic Science Self-Assessment (CBSSA) and submit a study plan by the first day of the course. The study plan must include the number of *UWorld QBank* practice questions scheduled for each week, completion dates for three passes through *First Aid for Step 1*, dates for a minimum of three practice tests, and the date of their Step 1 exam.

***Tracking progress:*** SASS tracks each student’s progress in their study plan using weekly, confidential, web-based surveys. Students are encouraged to ask questions and share concerns about their progress in the survey comment section. They may seek counseling at any time, whether they perceive a problem or simply desire to maximize their performance. Students are required to pass the USMLE Step 1 exam to pass the course.

The course director tracks individual survey responses and intervenes if students request assistance, are making slow progress, report practice exam scores below passing (194), or complete less than 50% of *Qbank* questions after the third week of the course. In addition, the course director reaches out periodically to all students, even those doing well, to provide motivation and encouragement. Two-week extensions are granted if a student is diagnosed with a significant medical condition (mental or physical) and are also considered for students who fail two or more practice tests in the last two weeks of the course. The decision for granting extensions requires agreement of the course director and the Associate Deans for Student Affairs.

Comprehensive Basic Science Self-Assessment (CBSSA) scores for 2017 and 2018, converted to Step 1 equivalent scores, and first time USMLE Step 1 Exam scores for UT Southwestern students for 2016, 2017 and 2018 (with national benchmark data) were obtained directly from the National Board of Medical Examiners (NBME) website. The percentage of students in a score range were interpolated from the NBME Score Histogram plots. Final exam averages were calculated from first-attempt final exam scores from the fifteen pre-clinical courses (not adjusted for scores on remedial exams). The difference between categorical data was determined using Chi-squared analysis and between means by Student’s T-test. Linear regression analysis was used to determine relationships between USMLE Step 1 Exam scores, average final exam scores for pre-clinical courses and results of the CBSSA exam. Finally, logistic regression analysis utilizing SAS Software Version 9.4 was employed to model factors that influenced the odds of specific levels of performance on the Step 1 exam.

## Results

From January 2017 to July 2018, 453 University of Texas Southwestern medical students completed the Step 1 Prep Course and sat for the Step 1 exam, and 446 had average final exam scores from the current curriculum. Seven students, whose pre-clinical course work was during the previous curriculum, are not part of this study. The probability of obtaining a Step 1 score below 210 rose from 1% in students with exam averages greater than 70%, to 6%, 8%, 17%, and 25% if they scored less than 70% in one, two, three, or more than three courses as noted in Table 1.

**Table 1.** Number of Course Final Grades <70 and Step 1 Scores

Number of Courses with Final Exam Score < 70	% Students with Step 1 Score < 210	Statistical Significance versus No courses below 210
**Zero**	1 %	--
**One**	6 %	p < 0.02
**Two**	8 %	p < 0.002
**Three**	17 %	p < 0.001
**Four or More**	25 %	p < 0.001

**Figure 1.**
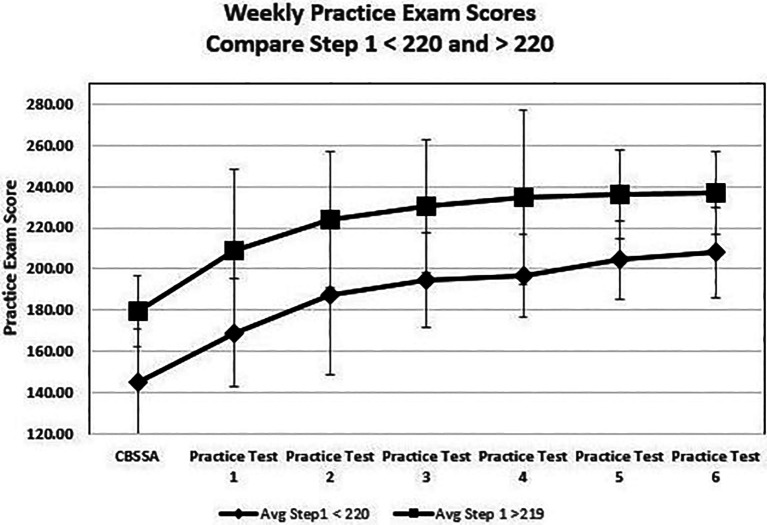
Weekly Practice Exams

The percentages of students failing the Step 1 exam were 11%, 6%, 1%, and 0% of students with final exam averages in the ranges 65%-69%, 70%-74%, 75%-79% and greater than 80%, respectively. The relationships between the initial CBSSA exam scores (designated as Week 0) and subsequent scores were analyzed by sequencing practice exams in the order in which they were taken (practice exam 1, 2, 3, etc..), since students were quite variable in the timing. The upper curve (squares) in Figure 1 illustrates the mean values and standard deviation of the CBSSA and practice exam scores throughout the course for students who scored greater 220 on their Step 1 exam; the lower curve (diamonds) plots results for those that scored below 220.

Note the significant improvement from the CBSSA exam to first practice exam followed by continued improvement on practice exams 2 and 3. Mean scores then plateaued in both groups. Students with Step 1 scores above 220 had significantly higher CBSSA scores than those with scores less than 220, and although both groups improved during the course, students scoring lower than 220 never reached practice exam performance of students who scored higher than 220.

***Subgroup analysis*** *:* The results of multiple linear regression are summarized in Table 2 and 3.

**Table 2.** Regression Statistics

Regression Statistics	
**Multiple R**	0.777049
**R squared**	0.060381
**Adj. R squared**	0.060100
**Standard Error**	11020783
**Observations**	441

**Table 3.** Summary of Regression Analysis

Variable	Coefficient	Standard Error	p-value	Lower 95 % Confidence Level	Upper 95 % Confidence Level
**Intercept**	68.92442	7.865246	4.21E-17	53.46611	84.38274
**Ave. Final Exam Scores**	0.241646	0.023539	2.67E-22	0.195382	0.287909
**CBSSA Exam**	1.543152	0.116532	6.66E-34	1.314121	1.7783

Average course final exam scores and CBSSA exam scores were significantly and directly associated with Step 1 scores. The multiple regression model predicted Step 1 scores with an R ^2^ value of 0.604, F value of 333.76, and an F significance < 0.001. Thus, measures of pre-clinical performance predicted about 60 % of the variability in Step 1 exam scores.

Logistic regression was then used to model the likelihood of a student scoring above and below 210 on Step 1. Students who scored below 210 increased exam scores with more weeks of practice, but were not affected by the total number of practice exams reported. The higher the first practice exam score, the higher the chance that the student would score greater than 210. For example, student with an initial practice score above 180 was 26 times more likely to score above 210 than a student below 180. D _max_, the score on the last practice exam minus the score on the initial practice exam, was inversely related to likelihood of scoring less than 210 on the step exam with a log Odds Ratio of 0.979. This calculates to about a 10 % decrease in the odds of obtaining a Step 1 score less than 210 for each 5 unit increase in D _max._ Stated alternatively, a student scoring less than 180 on the first practice exam could on average decrease the odds of scoring less than 210 from 26- to 18-fold if they increased practice exam scores 15 points during the course.

Data was remodeled to examine factors associated with obtaining a Step 1 exam greater than 220, a common screening value used to screen for residency interviews. The number of practice examinations did not significantly impact the likelihood of student scoring above 220 but a higher score on the initial practice examination and a larger D _max_ did. When compared to the students scoring less than 200 on the first practice examination, students scoring between 200 and 220 are 14.9 times, and those scoring above 220 are 55.3 times, more likely to score greater than 220 on the Step 1 exam compared to colleagues who scored less than 200. D _max_ was positively related with scoring above 220 with a log Odds Ratio for D _max_ of 0.0526 indicating student could double their odds of scoring greater than 220 on the Step exam if they increased their practice scores 15 points during the course.

**Figure 2.**
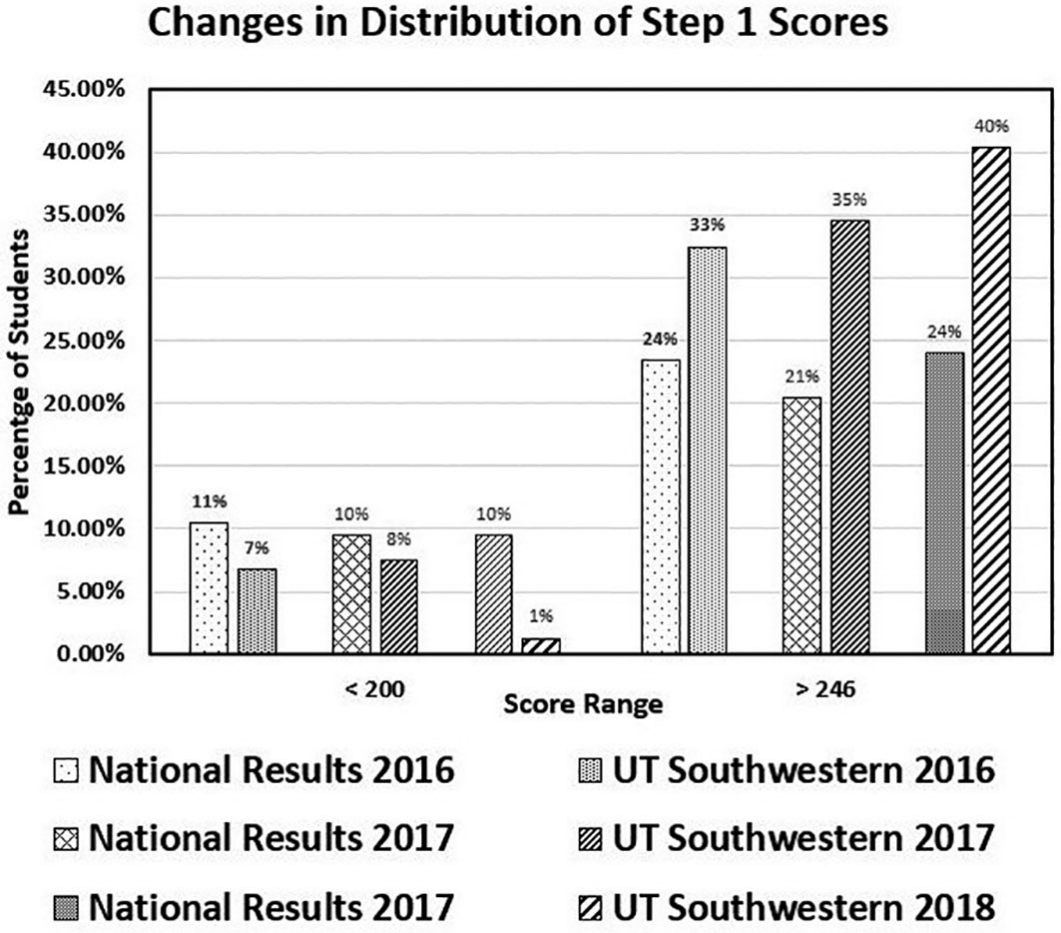
Comparison of UT Southwestern Step 1 Performance by Year

***Comparison of UT Southwestern performance with national benchmarks:*** The overall performance of the UT Southwestern students on the USMLE Step 1 exams was benchmarked with NBME-reported national outcomes from 2016 through 2018 in Figure 2.

The percent of students who scored below 200 and above 245 are plotted by year. For each year, UT Southwestern students had a lower percentage of scores below 200 and higher percentage of scores greater than 245 than the national mean. The percentage of students scoring greater than 245 increased from 33% in 2016 to 40% (compared to the national mean of 24%) in 2018. By 2018, only 1% of students scored below 200 compared with a national mean of 10%. The percent pass rates and mean scores for UT Southwestern students in 2016 and 2017 were essentially the same at 97% and 235, but in 2018, no student failed Step 1 (100% pass rate), and the mean score rose to 239.

UT Southwestern Human Research Protection Program (HRPP) determined that this project does not meet the definition of research under 45 CFR 46.102 and, therefore does not require IRB approval or oversight.

## Discussion

The UT Southwestern Step 1 Prep Course was designed to support students as they tackle their first high-stakes NBME licensure exam. The perception of the importance of the Step 1 exam by students is currently magnified beyond concern for licensure because residency program directors commonly use the Step 1 score to screen students for residency interviews ( Gauer and Jackson, 2017; Schwartz *et al.*, 2018; Federation, 2019). Considerable concern and anxiety are, therefore, generated amongst medical students who view the exam as a critical factor in obtaining the residency position of their choice ( Strowd and Lambors, 2010). The combination of this anxiety with poorly defined study strategies, faulty reasoning and poor wellness habits causes many students to struggle with their preparation. Many students relate that they do not know how to begin preparation for exam, decide on which resources to use in their preparation or even structure self-study time. All too often, they seek advice from inaccurate blogs, make poor assumptions based on erroneous data, and blindly follow advice from upper class students, which may or may not be pertinent to them. Physical and emotional health is often neglected because they fear that admitting to a problem is a “sign of weakness,” and evaluation and treatment sessions might cut into their limited study time. A strong case can therefore be made that students require support through this challenging period.

Since January 2017, second-year medical students in our current curriculum participate in the required Step 1 Prep Course. The course allows for considerable self-directed study while SASS provides guidance, support, and oversight during their study period. The course is well accepted by our medical students, who report that they feel emotionally supported during the prep period and that it fosters their success on the Step 1 exam: post-course surveys revealed that 70% thought the course was helpful versus 20% who did not perceive benefit. Most importantly, monitoring students identified those progressing slowly and allowed for helpful intervention before the exam was taken.

The data from this study has been quite informative as we assess and modify the Step 1 Prep Course. During the second year of the course, we increased email interaction, phone contact, and face-to-face consultation with students. At-risk students are now matched with an experienced upper-class student to aid with development of effective study strategies. In addition, students are now more liberally referred to the Student Wellness and Counseling center for testing and treatment of anxiety and stress disorders. The performance of students on the Step 1 exam reassure us that the current 18-month pre-clerkship curriculum and Step Prep Course prepared them for the exam. These data also validate findings in the literature that pre-clinical course performance and CBSSA exam scores are predictors of Step 1 performance ( Burk-Rafel, Santen and Purkiss, 2017; Giordano, Hutchinson, and Peppler, 2016; Bonasso *et al.*, 2015). The analysis of our data also generated useful cut-off values specific to our institution and showed that monitoring practice exams can be useful in identifying students who require assistance. For example, we now define a student with an initial CBSSA score lower than 170 as high-risk, but are less concerned if the student scores greater than 200 on their first practice exam. Likewise, students with higher initial CBSSA scores, but low first practice exam scores or poor progress on subsequent practice exams may require intervention. Moreover, whereas our finding that students in general did not benefit from study beyond 6 weeks is consistent with the findings of others ( Burk-Rafel, Santen, and Purkiss, 2017; Bonasso *et al.*, 2015; Kim and George, 2018), a subgroup of at-risk students did show improvement with extra weeks of dedicated study. This includes students with treatable medical and mental health problems. We therefore conclude that individual assessments using multiple parameters is warranted since no single factor reliably predicts Step 1 performance and note that the exact criteria for determining which students require advice and intervention must be determined within the context of each individual medical school’s curriculum and student population.

## Conclusion

The conclusions that can be made from this study are limited. It was a non-randomized study, and there were no control groups. In addition, the heterogeneity of a large medical school class and the nuances of managing a complex curriculum generated many hidden factors which cannot be controlled for or measured. Nonetheless, several conclusions can be made. First, Step 1 performance is strongly dependent on a strong knowledge base developed during pre-clinical courses. This influence had a lasting effect since low and high performers on course exams separated into two distinct groups, despite a common 6-week study period. A Step 1 Prep Course is not a substitute for mastering the core of medical knowledge to pass Step 1, but rather an adjunct to it. Second, these findings are not to be interpreted as evidence that at-risk students cannot obtain excellent scores on the Step 1 exam or that students who do well in pre-clinical courses are certain to avoid low scores. Many students either out- or underperformed their predicted score from curriculum performance data. Third, since the majority of progress occurred with the first 3 practice exams, decisions concerning interventions should be made before the midpoint of the prep course. Finally, although in general students do not benefit from extension of the study period, a subset of high-risk individuals benefited from a 2-week extension of the course. This primarily included students with medical and mental health problems amenable to treatment.

In summary, at UT Southwestern a structured Step 1 Prep Course is well-accepted by students and can serve in a supportive role in increasing student success on Step 1. Pre-clinical exam performance, practice exam scores at the beginning of the Step 1 preparation period and progress on practice exams over the study period correlate with Step 1 performance. Proactive utilization of these data during the Step 1 study period can be effective methods of identifying at-risk students and providing supportive interventions.

## Take Home Messages

• Step 1 performance is strongly dependent on a strong knowledge base developed during pre-clinical courses.
• Curriculum performance data is not a definite indicator that a student is at risk of underperforming on the Step 1 assessment.
• Successful curriculum performance should not be used to indicate if a student does not need guidance.
• Decisions of intervention during Step 1 preparation can and should be made before the half way mark of a 6 week Step prep course.
• A combination of a student’s self-study and the guidance of a mentor reduces failure rate for Step 1.

## Notes On Contributors

Arlene Sachs, PhD is Director of Student Academic Support Services, and is Assistant Professor of Psychiatry, UT Southwestern Medical Center, Dallas, Texas.

Blake Barker, MD, is Associate Dean of Students, and is Associate Professor of Internal Medicine, UT Southwestern Medical Center, Dallas, Texas.

Angela Mihalic, MD, is Dean of Medical Students, Associate Dean of Student Affairs, and is Professor of Pediatrics, UT Southwestern Medical Center, Dallas, Texas.

Dorothy Sendelbach, MD, is Distinguished Teaching Professor of Pediatrics and is Assistant Dean of Undergraduate Medical Education, UT Southwestern Medical Center, Dallas, Texas.

Carol Wortham, MEd, is Manager of Student Academic Support Services, UT Southwestern Medical Center, Dallas, Texas.

Robert Rege, MD, is Associate Dean for Undergraduate Medical Education,Associate Dean Continuing Medical Education, and is Professor of Surgery, UT Southwestern Medical Center, Dallas, Texas.
