# Supplementary material for: No Medical Student Left Behind: A Study to Examine the Effects of Structured Step 1 Support within our Curriculum
Source: MedEdPublish (2016). 2019 Sep 12;8:171. [Version 1] doi: 10.15694/mep.2019.000171.1 (PMC10712451; doi:10.15694/mep.2019.000171.1)
Supplement: Peer review history

# Reviewer report: Reviewer response for version 1

**DOI:** 10.21956/mep.19897.r30115

**Published:** 2020-05-12

**Authors:** Richard Hays

**Affiliations:** 1 James Cook University

This review has been migrated. The reviewer awarded 4 stars out of 5 This paper reports an approach at one medical school to the challenging issue of increasing the success of Step 1 candidates in the national Step 1 exam. It reads well, uses appropriate methods and reports success in getting students 'over the line'. This is not a surprise - students love practice exams, and if the practice exams are well-aligned with the external exam, success should predict success. This of course requires a lot of work by the faculty to generate a sound examination with the same blueprint and item quality. For that, the authors are to be congratulated. However, the paper also raises important issues about assessment practice design and philosophy and the impact on learners. Should progress be based on more than just a single test score when specific exam coaching achieves 100% progress? What is the impact on other exams and learning throughout the curriculum? Was this a retreat to 'teaching for the test"? Did the practice exam approach look similar to progress testing testing, with Step 1 calibration but broader coverage to include other, perhaps non-core, topics? Some comment on these issues might add interest, but the paper should be of interest to other medical schools whose students face national licensing exams.

Reviewer Expertise:

NA

No decision status is available

# Reviewer report: Reviewer response for version 1

**DOI:** 10.21956/mep.19897.r30117

**Published:** 2019-11-10

**Authors:** Ken Masters

**Affiliations:** 1 Sultan Qaboos University

This review has been migrated. The reviewer awarded 4 stars out of 5 An interesting paper on a study to examine the effects of structured step 1 support within a curriculum.The paper is well-written, beginning with a good overview of the problem and a comprehensive description of the course. A danger of an intervention course like this is that it threatens to become simply a teaching-for-the-exam exercise, in which (through the practice exams) students are essentially resitting the exam until they pass. In this course, though, the institution has taken a much broader approach than simplistic exam preparation, and has simultaneously addressed all aspects of the students’ work, increasing their students’ overall preparedness, and their chances of becoming better health professionals.Two small points:• The Discussion mentions data from post-course surveys. These data (and other numbers about them) should be given in the Results, and the surveys need to be described in the Methods, otherwise they should not be discussed here.• The limitations are mentioned only in the Conclusion, and should really be at the end of the Discussion.

Reviewer Expertise:

NA

No decision status is available

# Reviewer report: Reviewer response for version 1

**DOI:** 10.21956/mep.19897.r30116

**Published:** 2019-09-18

**Authors:** Felix Silwimba

**Affiliations:** 1 University of Lusaka

This review has been migrated. The reviewer awarded 5 stars out of 5 this article is informative and educative. the authors have adequately shared their experiences in student preparations for step 1 licentiate examinations.

Reviewer Expertise:

NA

No decision status is available
